# qPCR assays to quantitate tRNApyl and pylRS expression in engineered cell lines

**DOI:** 10.1371/journal.pone.0216356

**Published:** 2019-05-09

**Authors:** Andrew Garcia, Gargi Roy, Christine Kiefer, Susan Wilson, Marcello Marelli

**Affiliations:** Department of Antibody Discovery and Protein Engineering, AstraZeneca, Gaithersburg, MD, United States of America; Scripps Research Institute, UNITED STATES

## Abstract

Non-natural amino acids (nnAA) contain unique functional moieties that greatly expand the available tool set for protein engineering. But incorporation of nnAAs requires the function of an orthogonal aminoacyl tRNA synthetase/tRNA pair. Stable cell lines expressing these components have been shown capable of producing gram per liter levels of antibodies with nnAAs. However, little has been reported on the genetic makeup of these cells. To gain a better understanding of the minimal requirements for efficient nnAA incorporation we developed qPCR methods for the quantitation of the key components. Here we describe the development of qPCR assays for the quantification of tRNApyl and pylRS. qPCR was chosen because it provides a large dynamic range, has high specificity for its target, and is a non-radioactive method used routinely for cell line characterization. Designing assays for tRNAs present challenges due to their short length (~72 nucleotides) and high secondary structure. These tRNA assays have a ≥ 5 log dynamic range with the tRNApyl assays being able to discern the mature and unprocessed forms of the tRNApyl. Cell line analysis showed tRNApyl was expressed at higher levels than the CHO-K1 endogenous Met and Phe tRNAs and that >88% of tRNApyl was the mature form.

## Introduction

Over the last ten years bioconjugates have emerged as a promising new class of medicines that offer improved performance of therapeutics. Bioconjugations have been used extensively to improve the half-life of proteins (e.g. PEGylation) and more recently for the construction of antibody drug conjugates (ADCs) [1,2]. ADCs are a promising new class of engineered biotherapeutic that combines the targeting specificity of antibodies with potent cytotoxins for the treatment of cancers. Early ADCs were generated by targeting cysteine thiols, or the primary amine of lysine for payload conjugation. However, this strategy generated heterogenous ADCs that have shown variable payload stability and reduced therapeutic effect [3,4]. To address these limitations technologies have emerged that enable site-specific modifications of target proteins to better control the homogeneity and stability of the products. One of the most attractive methods involves the site-specific incorporation of non-natural amino acids (nnAAs) containing moieties that enable biorthogonal conjugation chemistries [2,5–11]. Biorthogonal conjugation methods including oxime, Diels-Alder and click cycloaddition have all shown efficient conjugate formation and improved stability of the conjugates over the commonly used thiol-maleimide conjugations.

The drawback to using nnAAs lies in the production of proteins containing these nnAAs. Expression systems in *E*. *coli*, yeast, mammalian cells (CHO and HEK293), and cell free expression systems have been developed and shown efficiency in nnAA incorporation [6, 8, 12–16]. Of these, mammalian cell expressions have the distinct advantage that they conform with conventional fermentation processes, contain a reliable glycosylation pattern, and are generally devoid of endotoxin. Thus, great effort has been dedicated to developing cell lines that are capable of high titer expression of nnAA containing proteins. Stable cell lines expressing orthogonal aminoacyl tRNA synthetase/tRNA pairs have shown yields exceeding 1g/L [7, 17]. However, a good understanding of the minimal expression requirements of aaRS and its tRNA to enable efficient amber suppression is lacking. While the detection and quantification of aaRS expression is straightforward, the same cannot be said of tRNA. The measurement of tRNA is challenging due to its short size, and extensive secondary structure which restricts available qPCR probe sites. In addition, mature cytosolic tRNA, which averages 72 nucleotides is derived from a larger precursor molecule that undergoes 3’ and 5’ processing, and in eukaryotic cells is further modified with a 3’ trinucleotide (CAA) to generate a functional tRNA [18]. Gel based methods requiring hybridization have been used to quantify the expression and assess the level of maturation of tRNAs [19–21]. While this method allows for the quantitation of aminoacylated, free, unprocessed and mature tRNA, it requires radioactive probes and is of low throughput. Molecular approaches to quantify specific tRNAs like four-leaf clover qPCR have been developed, but require large amounts of RNA, has a limited dynamic range, and requires multiple enzymatic steps [22].

We have developed a CHO-based expression system for the expression of biotherapeutics containing nnAAs [7]. The cells utilize the pyrrolysine tRNA synthetase (pylRS), derived from *Methanosarcina mazei*, with specificity for a nnAA and its cognate pyrrolysine tRNA (tRNApyl) which directs the nnAA to amber codons in the gene of interest [11]. However, the identification of engineered cells capable of efficient nnAA incorporation required extensive functional screening using reporter constructs to assess amber suppression. The development of these cell lines showed that high tRNA gene copy numbers were needed to achieve the desired nnAA incorporation efficacy in both transient and stable cell lines. To understand the requirements for efficient nnAA incorporation we utilized a qPCR method useful for the quantitation of these genes. The assays were developed to assess both the DNA copy number and RNA expression levels of pylRS and its cognate tRNApyl in cell lines capable of amber suppression. This method provides a necessary tool for the characterization of these cells and the determination of the minimum requirements for efficient amber suppression in host cells.

## Materials and methods

### RNA and DNA isolation

CHO cells selected for RNA and DNA isolation were grown in suspension in CD-CHO medium (LifeTechnologies) to a viable density of 1–2×10^6^ cells/mL. All cultures were grown at 37°C in shake flasks, in an environment controlled shaking incubator maintaining 6% CO_2_, and 80% humidity (Multitron HT, Infors). Culture volumes containing 5×10^6^ cells were subjected to centrifugation, medium was discarded, and pellets frozen at -80°C. Total RNA (tRNA, mRNA and rRNA) was isolated from frozen cell pellets consisting of 5×10^6^ CHO cells using the mirVANA miRNA Isolation kit (Ambion) following the manufacturer’s recommendations. Contaminating DNA was removed from the purified RNA samples using a DNA removal kit (Ambion). Genomic DNA (gDNA) was isolated from frozen cell pellets using the AllPrep DNA/RNA Mini Kit (Qiagen). For each gDNA isolation 5×10^6^ cells were lysed in 600μl lysis buffer and purified following the manufacturer’s instructions.

### Plasmid DNA

pCEP4-pylRS, pylRS gene was cloned under control of the CMV promoter in pCEP4 (LifeTechnologies). pBS-tRNApyl, pBluescript SKII (Stratagene) containing a the tRNApyl gene.

### Primer design and oligomers

The primers and probes used in this study were custom designed based on available gene sequences. tRNApyl oligo (72bp = 1.35×10^10^ copies/ng oligo), tRNApyl unprocessed oligo (87bp = 1.12×10^11^ copies/ng oligo), CHO-K1 tRNAmet oligo (72bp = 1.35×10^10^copies/ng oligo) and CHO-K1 tRNAphe oligo (73bp = 1.33×10^10^ copies/ng oligo). The primers and probes for CHO-K1 Beta-2 microglobulin (CHO-K1 B2M) were designed by entering a single CHO-K1 B2M exon from GenBank NW_006871970 into ABI’s “Custom Taqman Assay Design Tool.” All DNA oligos were synthesized by IDT and DNA probes were synthesized by ABI. The 18S rRNA (18S) assay was purchased from ABI (Hs999999_01) and used at 1X final concentration in qPCR assays and produces an amplicon of 187bp. No primer sequence information is available for the 18S purchased from ABI.

### qPCR of gDNA

gDNA samples were analyzed using the Taqman Fast Universal PCR Master Mix (ABI). All assays were performed on the 7900HT Fast Real-Time PCR instrument using a “fast 96 well plate.” Thermocycler conditions were 1 cycle of 95°C for 20sec followed by 40 cycles of 95°C for 1sec and 60°C for 20sec. Each reaction contained 10ng gDNA, 1μM forward primer, 1μM reverse primer and 0.25μM probe. All samples were analyzed in triplicate.

### qRT-PCR of RNA

RNA samples were analyzed by one-step qRT-PCR method using the Express One-Step SuperScript qRT-PCR with Pre-mixed ROX kit (LifeTechnologies). Reverse Transcription was performed using gene specific primers. For tRNA qRT-PCR, purified total RNA, primers and water were mixed and incubated at 80°C for 5 min, then allowed to cool to room temperature. Buffers and SuperScript qRT were added prior to the qRT-PCR incubations. All assays were performed on the 7900HT Fast Real-Time PCR instrument using a “fast 96 well plate” with each reaction containing 5ng RNA in a 20μl reaction volume. Thermocycler conditions were 1 cycle of 60°C for 15min for Reverse Transcription then 1 cycle of 95°C for 20sec followed by 40 cycles of 95°C for 1sec and 60°C for 20sec. All samples were analyzed in triplicate.

### Cell line construction

Cell lines stably expressing pylRS and tRNApyl were constructed as described in [7]. Briefly, Chinese hamster ovary cells (CHO), adapted to serum free suspension growth, were transfected with the plasmid pCLD-RS-18xtRNA, encoding pylRS under control of the CMV promoter, 18 tandem repeats of the tRNApyl under control of the U6 snRNA promoter, and a puromycin resistance cassette. Transfected cells were grown in CD-CHO medium containing puromycin and survivors functionally characterized for amber suppression. To do this, mRNA encoding an RFP-GFP fusion, which contains an amber stop codon interrupting the RFP and GFP coding regions, was transfected into these cells. With this reporter all transfected cells express RFP (RFP+/GFP-), but only cells capable of amber suppression express the RFP-GFP fusion (RFP+/GFP+) that can be readily observed and quantified by flow cytometry. The ratio of GFP:RFP may be indicative of the amber suppression efficacy in each of the cell lines. Thus, cells with the highest GFP:RFP ratios were selected for characterization.

## Results

### Testing assays for nnAA components on plasmid DNA

**Fig 1:**
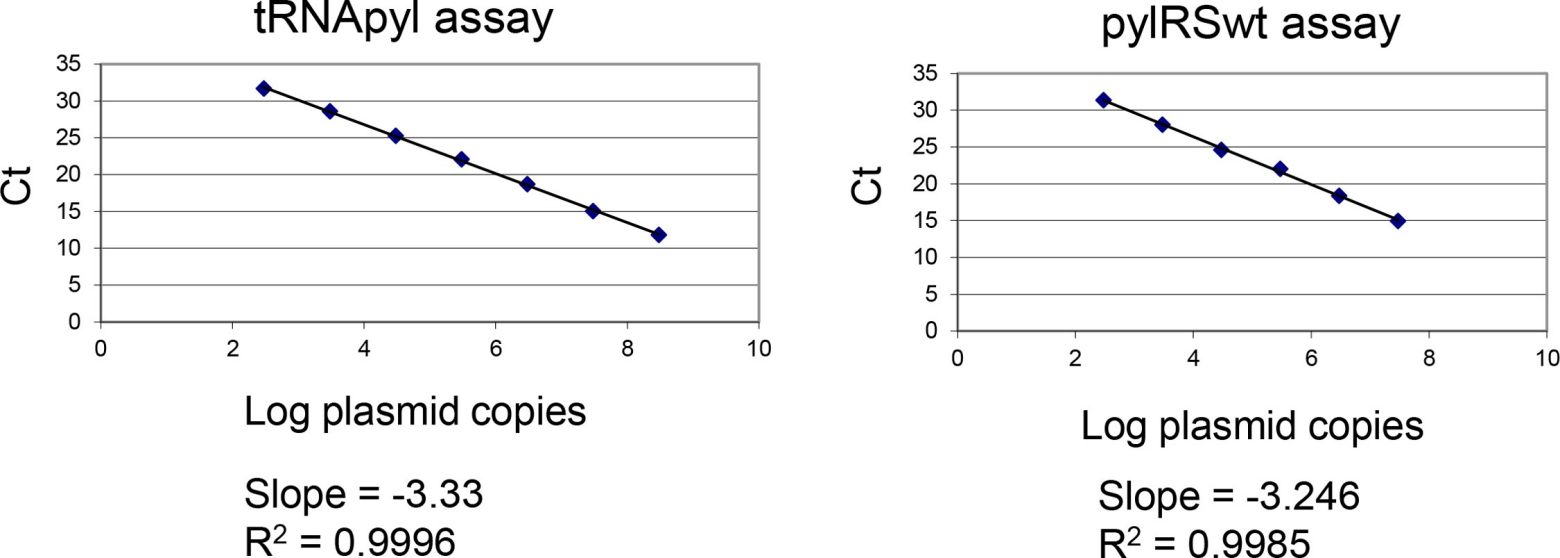
qPCR assay performance on plasmid DNA. The tRNApyl and pylRSwt qPCR assays were tested with a tenfold serial dilution of their respective plasmids spanning 300–3×10^8^ copies. Graphing the Ct versus the log of plasmid copies demonstrates a linear range of 300–3×10^8^ copies for tRNApyl and 300–3×10^7^ copies for pylRSwt. A simple linear regression shows R^2^ values approaching 1 and slope values close to -3.3.

We set out to establish assays to determine the gene copy number and relative expression of pylRS and tRNApyl genes in engineered cell lines selected for their activity. While the assay for pylRS is straightforward, the high secondary structure of tRNA posed a challenge for qPCR. Thus, we began by developing a qPCR assay using plasmid DNA encoding the tRNApyl or pylRS as a template. This allowed us to test conditions without the confounding effect of tRNA structure. For each assay gene specific primer sets (Table 1) were used with a titration of the template DNA. The data shows a linear relationship between Ct and the log of plasmid copies ranging from 300 to 3×10^8^ copies for the tRNApyl gene, and 300–3×10^7^ for pylRS (Fig 1). For reference, 1ng of the pCEP4-pylRS (11,545bp) plasmid represents 8.44×10^7^ copies of the pylRS gene; and 1ng of pBS-tRNApyl (3,287bp) contains 2.97×10^8^ copies of the tRNA gene. In each case we observed a coefficient of determination (R^2^) approaching 1 and a slope approaching of -3.3. These data show that the qPCR assay has acceptable efficiency through this linear range and indicates that the primer/probe sets are adequate for the qPCR of these genes.

**Table 1:** Primers, probes, DNA oligo templates.

tRNApyl	Forward	GGAAACCTGATCATGTAGATCG
	Reverse	GGAAACCCCGGGAATC
	Probe	FAM- ACCCGGCTGAACGGATTTAGAGTC -TAMRA
tRNApyl unprocessed*	Reverse	CGCACTTGTCCGGAAAC
pylRSwt	Forward	ATCAAGCACCATGAGGTGTC
	Reverse	ACCGCTTGCAGGTCTTTC
	Probe	FAM- CCAAGATCTACATCGAGATGGCCTGC -TAMRA
CHO-K1 tRNAmet	Forward	GCAGAGTGGCGCAGC
	Reverse	GTTTCGATCCATCGACCTC
	Probe	FAM- GGGCCCAGCACGCTTCC -TAMRA
CHO-K1 tRNAphe	Forward	GAAATAGCTCAGTTGGGAGA
	Reverse	TGAAACCCGGGATCGAA
	Probe	FAM- CGTTAGACTGAAGATCTAAAGGTCCCTGG -TAMRA
CHO-K1 B2M	Forward	CGAGCTGTTGAAGAATGGAAAGAAG
	Reverse	CGTGTGAGCCAAAAGATAGAAAGAC
	Probe	FAM- ACAAAGTCGAGCTGTCAGATCT-NFQ
tRNApyl	cDNA	CGGAAACCCCGGGAATCGAACCCGGCTGAACGGATTTAGAGTCCATTCGATCTACATGATCAGGTTTCC
tRNApyl unprocessed	cDNA	AAAAAAACCGCACTTGTCCGGAAACCCCGGGAATCTAACCCGGCTGAACGGATTTAGAGTCCATTCGATCTACATGATCAGGTTTCC
CHO-K1 tRNAmet	cDNA	TAGCAGAGGATGGTTTCGATCCATCGACCTCTGGGTTATGGGCCCAGCACGCTTCCGCTGCGCCACTCTGCT
CHO-K1 tRNAphe	cDNA	TGCTGAAACCCGGGATCGAACCAGGGACCTTTAGATCTTCAGTCTAACGCTCTCCCAACTGAGCTATTTCAGC

*tRNApyl and tRNApyl unprocessed use the same forward primer and probe.

### Testing tRNA assays on DNA oligos

**Fig 2:**
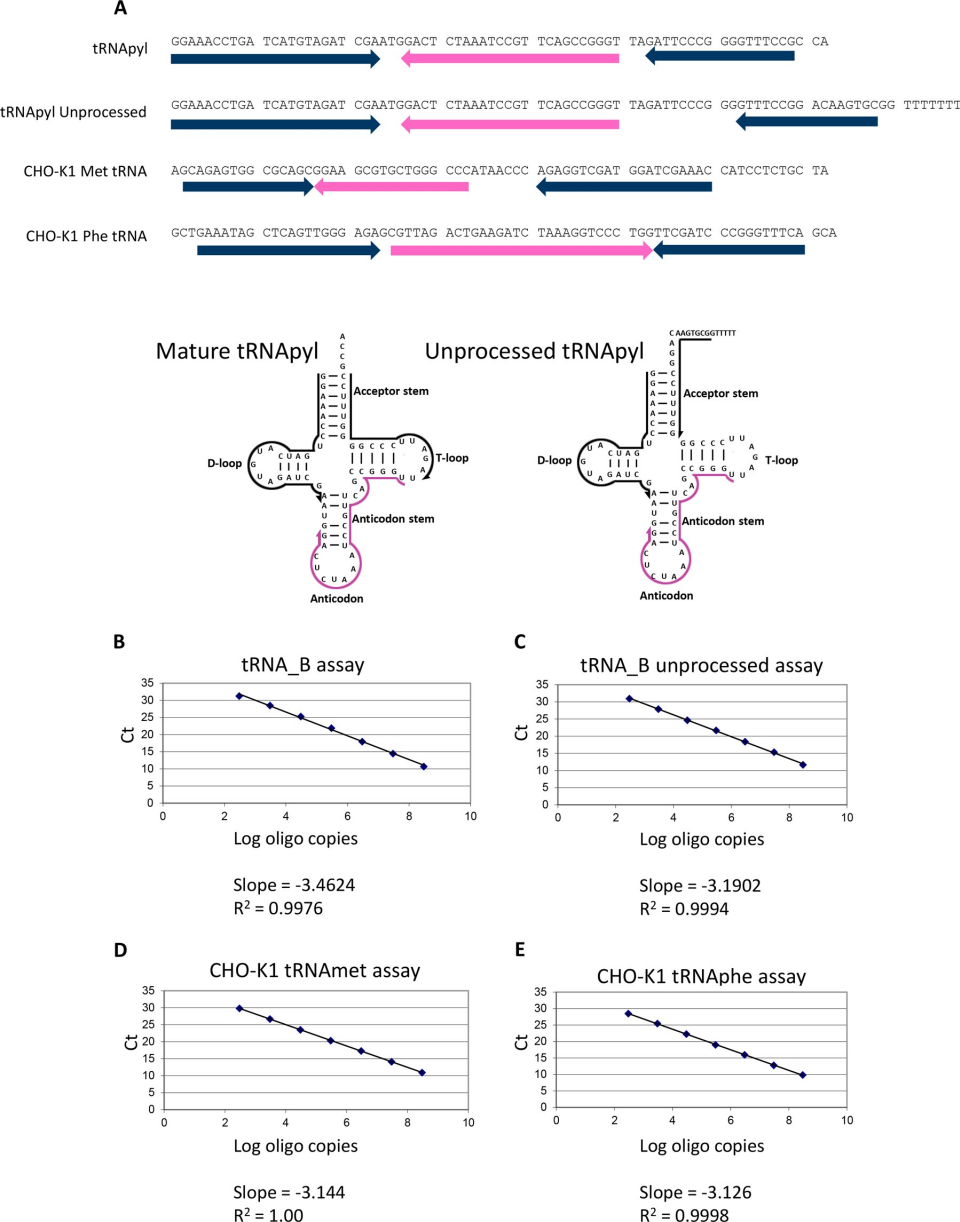
qPCR assay performance on single stranded DNA oligos corresponding to their target cDNA. (A) Sequence of DNA oligos with the qPCR primers in blue and the qPCR probes in red. 5’-3’ orientation is shown by the direction of the arrows. The position of the primers and probe for both mature and unprocessed tRNApyl are shown on the cloverleaf schematic of the tRNA. The primers for mature tRNApyl quantify total tRNApyl (mature and unprocessed). The reverse primer for unprocessed tRNApyl is specific for this species. (B) tRNApyl assay with the tRNApyl DNA oligo. (C) tRNApyl unprocessed assay with the tRNApyl unprocessed DNA oligo. (D) CHO-K1 tRNAmet assay with the CHO-K1 tRNAmet DNA oligo. (E) CHO-K1 tRNAphe assay with the CHO-K1 tRNAphe DNA oligo. The qPCR assays were tested using a tenfold serial dilution of their respective DNA oligo spanning 300–3×10^8^ copies. Plotting the Ct versus the log input of DNA oligo copies show that all qPCR assays have a linear range of 300–3×10^8^ with R^2^ values approaching 1 and slopes near -3.3.

Next, we tested the qPCR assay using DNA oligos as templates because they are expected to exhibit secondary structure consistent with cellular samples. In addition, tRNA undergoes extensive processing to its mature and active form. Thus, DNA oligos encoding the cDNAs of fully processed mature tRNApyl and the unprocessed tRNApyl were synthesized and used as templates using the primer pairs and probes described above (Fig 2A). Synthesized DNA oligos were the only materials available that we could control for quantity and sequence and are representative of a 100% efficient reverse transcription reaction. Assays for endogenous CHO-K1 tRNAmet and tRNAphe were designed to gauge expression levels of tRNApyl relative to natively expressed tRNAs. CHO-K1 tRNAmet and tRNAphe were chosen for their limited codon representation, specifically tRNAmet has a single codon, ATG, and the tRNAphe has two codons, TTT and TTC. As previously described, the qPCR assays were conducted against a ten-fold serial dilution of DNA oligo spanning 300–3×10^8^ copies. All four tRNA assays showed acceptable slope and R^2^ values with linearity from 300–3×10^8^ DNA oligo copies (Fig 2B–2E).

It should be noted that the amplicon for the tRNApyl assay is contained in both the tRNApyl and tRNApyl unprocessed DNA oligos and will amplify both mature and unprocessed tRNApyl (total tRNApyl). To examine the specificity of the tRNApyl and tRNApyl unprocessed assays, both assays were run against a ten-fold serial dilution of tRNApyl and tRNApyl unprocessed DNA oligos spanning 300–3×10^8^ copies of DNA oligo (Table 2). The assay for total tRNApyl yielded similar Ct values for both the tRNApyl and tRNApyl unprocessed DNA oligos. On the other hand, the tRNApyl unprocessed assay, shows a significant difference in Ct values between the tRNApyl and tRNApyl unprocessed DNA oligos. The increase in Ct of 13 corresponds to a reduction in sensitivity of 8000-fold, demonstrating that the tRNApyl unprocessed assay is relatively insensitive to mature tRNApyl. Mature tRNApyl levels were therefore calculated as the difference between tRNApyl and tRNApyl unprocessed.

**Table 2:** Specificity of tRNApyl and tRNApyl unprocessed qPCR assays on single stranded DNA oligo.

	**tRNApyl**		**tRNApyl unprocessed**	
	**assay**		**assay**	
**tRNApyl**	Mean	Std Dev	Mean	Std Dev
**cDNA oligo**	Ct	Ct	Ct	Ct
3x10E8 copies	10.71	0.02	24.28	0.12
3x10E7 copies	14.47	0.09	27.84	0.02
3x10E6 copies	17.98	0.03	31.13	0.25
3x10E5 copies	21.90	0.15	34.71	0.23
3x10E4 copies	25.27	0.16	37.52	0.08
3x10E3 copies	28.47	0.11	ND	ND
3x10E2 copies	31.26	0.08	ND	ND
H2O	ND	ND	ND	ND
	**tRNApyl**		**tRNApyl unprocessed**	
	**assay**		**assay**	
**tRNApyl unprocessed**	Mean	Std Dev	Mean	Std Dev
**cDNA oligo**	Ct	Ct	Ct	Ct
3x10E8 copies	10.65	0.40	11.64	0.55
3x10E7 copies	14.93	0.22	15.29	0.05
3x10E6 copies	17.97	0.08	18.35	0.01
3x10E5 copies	21.75	0.21	21.67	0.09
3x10E4 copies	25.16	0.15	24.64	0.05
3x10E3 copies	28.35	0.26	27.88	0.02
3x10E2 copies	31.14	0.26	30.93	0.06
H2O	37.99	1.41	ND	ND

ND, not detected

### Testing assays on cell line samples

Having established a quantitative qPCR assays for both tRNApyl, tRNApyl unprocessed and pylRS on DNA templates, we next set out to establish the conditions for quantifying RNA derived from cellular material derived from a cell line previously characterized for amber suppression activity [7]. For expression analysis this requires the establishment of an RT step to convert RNA to cDNA prior to amplification and DNase treatment to remove contaminating genomic DNA. Gene specific primers were used for reverse transcription since the tRNAs have no binding site for oligo dT, and random hexamers do not transcribe all the required tRNA sequences for PCR amplification. As previously mentioned the high secondary structure of tRNA was expected to present a challenge. Indeed, RT-PCR reactions of cellular derived material showed equal values for both mature and unprocessed tRNApyl. The observation that the majority of the tRNApyl was in unprocessed form was contrary to our expectations and we hypothesized that the secondary structure of the mature tRNApyl was inhibiting efficient primer binding. To denature the secondary structure of the tRNApyl prior to RT, an 80°C incubation step was implemented to allow primer annealing and reverse transcription. Prior to the inclusion of the 80°C incubation step, the unprocessed form constituted almost 100% of tRNApyl signal obtained. However, after the 80°C incubation step it accounted for only 10% of tRNApyl signal. This change in percentage of signal from the unprocessed form suggests that the reverse transcriptase step is efficient. The final assay for RNA is a one-step RT-qPCR and the data shown is reported as a ratio relative to 18S ribosomal RNA (18S). For the purposes of our RNA assays we chose to use a single gene, 18S, for normalization. There is always the concern that cell treatment will alter expression levels of your normalizing gene however, in our experience, the high expression of 18S provides a buffer against such variations. Having multiple normalizers (ie GAPDH and 18S) would require extensive effort to make sure that the expression levels of the normalizers are not being differentially impacted by cell treatments. Throughout this manuscript RNA and tRNA expression levels are referred to as expression ratios that are calculated using the following formula:

$$Expression\;Ratio=2^{\left(-\Delta ct\right)}\;where\;\;\;\Delta Ct=(Target\;Mean\;Ct)-(18S\;Mean\;Ct)$$

**Fig 3:**
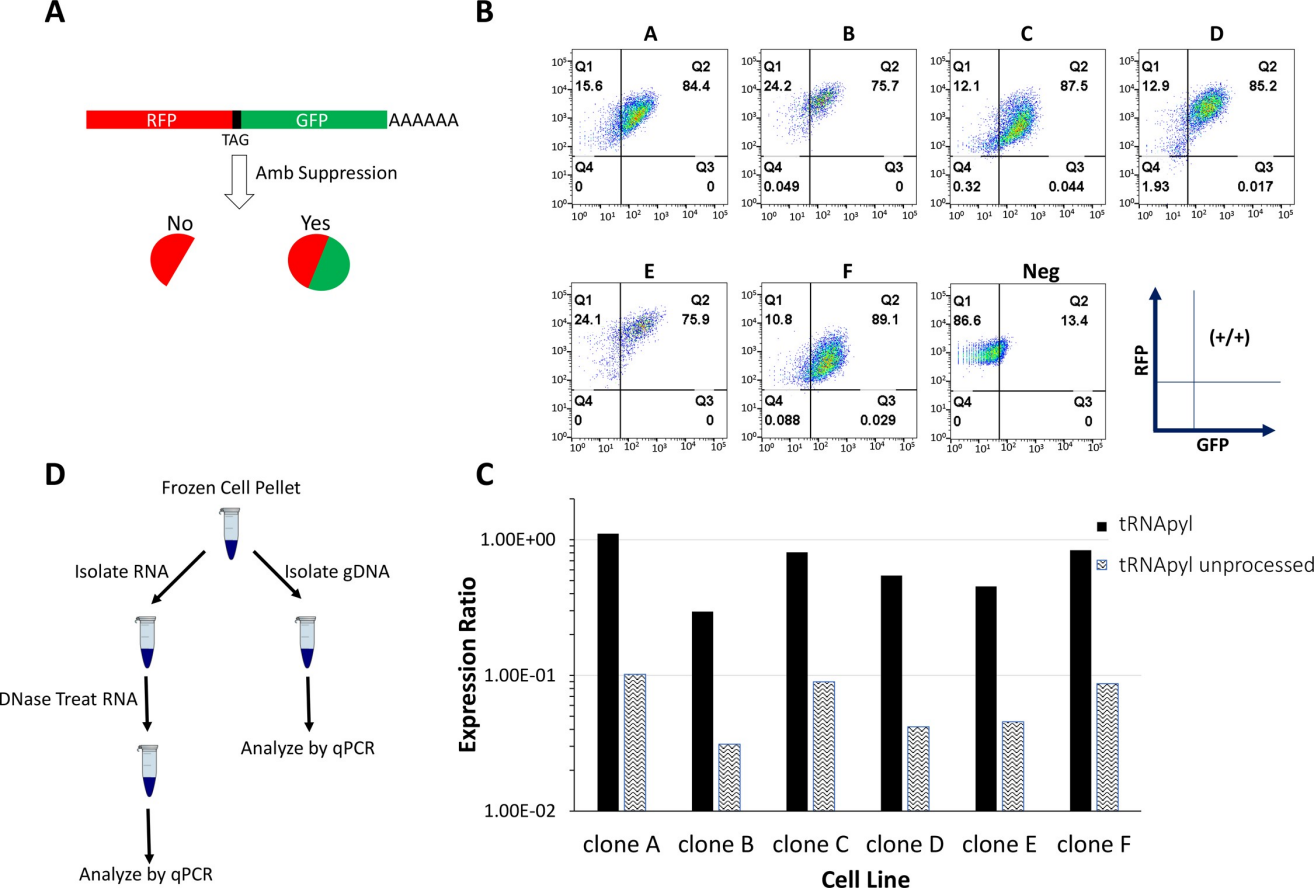
Quantifying RNA expression levels of unprocessed and mature tRNApyl in cell lines. (A) A reporter mRNA construct was designed to assess amber suppression activity. The reporter encodes an RFP-GFP fusion interrupted by an amber stop codon. All transfected cells show RFP expression, but only amber suppression competent cells express the RFP-GFP fusion. (B) Six clones isolated from an amber suppression competent host were transfected with the RFP-GFP mRNA and exposed to nnAA. Cells were grown for 24 h. and fluorescence expression examined by flow cytometry. Six positive clones showing RFP and GFP signals were identified. A cell line showing no GFP expression is shown for comparison (Neg). (C) RNA from the six amber suppression competent cell lines (A-F) was examined by the tRNApyl and tRNApyl unprocessed assays to quantify the extent of tRNApyl processing. (D) A schematic representation of the workflow for isolating and analyzing nucleic acids from cell lines. The data show that mature tRNApyl constitutes >88% of the tRNApyl forms present in the samples and indicate efficient processing of the tRNApyl in CHO cells.

**Fig 4:**
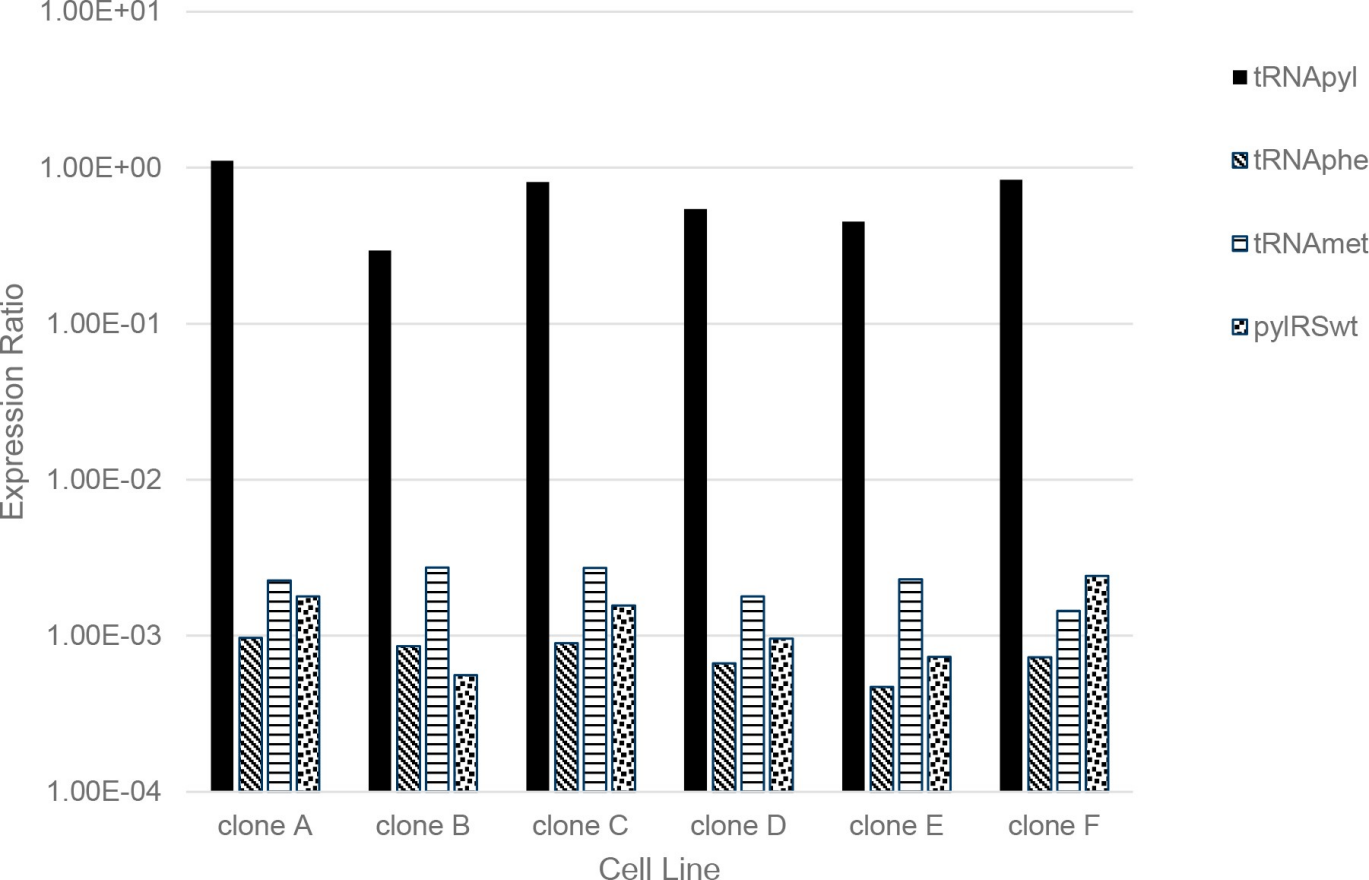
Quantifying RNA expression levels of native CHO-K1 tRNAs, tRNApyl and pylRSwt in engineered cell lines. RNA from six stable cell lines expressing pylRS/tRNApyl were assayed for endogenous tRNAphe, tRNAmet, as well as tRNApyl and pylRSwt. The data demonstrate that pylRSwt is expressed at comparable levels to the endogenous CHO-K1 tRNA. tRNApyl is expressed at ~1000-fold higher levels than endogenous tRNA.

Having established an effective method for measuring both mature and unprocessed tRNApyl total RNA we set out to test the assay on material derived from cells. To do this we isolated RNA from six different clonal cell lines derived from a host capable of amber suppression [7]. Each subclone was initially characterized for amber suppression activity by transfection of an mRNA construct encoding RFP-GFP containing an amber codon between the two fluorescent proteins (Fig 3A). The transfected cells were exposed to nnAA and the fluorescent proteins quantified by flow cytometry (Fig 3B). All transfected cells express the RFP reporter, but only cells capable of amber suppression express an RFP-GFP fusion. The ratio of GFP:RFP may be indicative of the pylRS/tRNA system activity. Thus, six clones (A-F), positive for amber suppression, were selected to examine the tRNA and pylRS content. The data show that these stable cell lines have high expression levels of the tRNApyl with ratios relative to 18S ranging from 0.29 to 1.11 and with approximately 90% of the tRNApyl in its mature form (Fig 3C and Table 3). Interestingly, endogenously expressed tRNAphe show expression ratios between 4–9×10^−4^ and tRNAmet between 1.44–2.73×10^−3^ (Fig 4 and Table 4). These levels are as much as 2000-fold lower than tRNApyl levels indicating that our engineered cells are high expressers of the tRNApyl which may be necessary to enable efficient amber suppression with nnAAs.

**Table 3:** Quantifying RNA expression levels of unprocessed and mature tRNApyl in cell lines.

	tRNApyl assay			tRNApyl unprocessed assay				
	Mean	Std Dev	Expression	Mean	Std Dev	Expression	tRNApyl	
Cell Line	Ct	Ct	Ratio	Ct	Ct	Ratio	Mature	Unprocessed
clone A	17.27	0.25	1.11E+00	20.72	0.34	1.02E-01	90.8%	9.2%
clone B	18.56	0.21	2.95E-01	21.80	0.27	3.11E-02	89.5%	10.5%
clone C	16.84	0.08	8.07E-01	20.02	0.26	8.96E-02	88.9%	11.1%
clone D	17.63	0.28	5.45E-01	21.33	0.40	4.18E-02	92.3%	7.7%
clone E	17.13	0.23	4.53E-01	20.44	0.34	4.55E-02	90.0%	10.0%
clone F	16.57	0.12	8.36E-01	19.83	0.17	8.69E-02	89.6%	10.4%

tRNA expression ratios are reported relative to 18S rRNA.

**Table 4:** Quantifying RNA expression levels of native CHO-K1 tRNAs, tRNApyl and pylRSwt in cell lines.

	**tRNApyl assay**			**tRNAphe assay**			**tRNAmet assay**		
	Mean	Std Dev	Expression	Mean	Std Dev	Expression	Mean	Std Dev	Expression
**Cell Line**	Ct	Ct	Ratio	Ct	Ct	Ratio	Ct	Ct	Ratio
clone A	17.27	0.25	1.11E+00	27.44	0.47	9.67E-04	26.21	0.36	2.27E-03
clone B	18.56	0.21	2.95E-01	26.99	0.39	8.55E-04	25.31	0.33	2.73E-03
clone C	16.84	0.08	8.07E-01	26.66	0.12	8.98E-04	25.06	0.35	2.72E-03
clone D	17.63	0.28	5.45E-01	27.30	0.04	6.67E-04	25.88	0.40	1.79E-03
clone E	17.13	0.23	4.53E-01	27.04	0.19	4.71E-04	24.75	0.14	2.30E-03
clone F	16.57	0.12	8.36E-01	26.73	0.13	7.29E-04	25.74	0.45	1.44E-03
	**pylRSwt assay**			**18s assay**					
	Mean	Std Dev	Expression	Mean	Std Dev				
**Cell Line**	Ct	Ct	Ratio	Ct	Ct				
clone A	26.55	0.27	1.79E-03	17.42	0.15				
clone B	27.60	0.08	5.58E-04	16.80	0.02				
clone C	25.86	0.18	1.56E-03	16.54	0.10				
clone D	26.78	0.26	9.58E-04	16.75	0.22				
clone E	26.40	0.13	7.32E-04	15.98	0.04				
clone F	25.00	0.07	2.42E-03	16.31	0.10				

tRNAphe, tRNAmet, tRNApyl, and pylRSwt expression ratios are reported relative to 18S rRNA.

### DNA copy number

Complementary assays to quantify the gene copy number of pylRS using qPCR assays were developed to gain further insight into the content of these engineered cells and examine the relationship between DNA copy number and RNA expression levels of the exogeneous genes. Standard qPCR assays were used for both tRNApyl and pylRS using the same primer sets described above. In addition, a primer set specific for CHO-K1 B2M was synthesized to serve as a standard for genomic copy quantitation (and assuming 2 copies of CHO-K1 B2M per genome). In this text DNA copy number calculations are referred to as Copy Number and are determined with the following formula:

$$Copy\;Number=2^{\left(-\Delta ct\right)}\;where\;\;\;\Delta Ct=(Target\;Mean\;Ct)-(CHOK1\;B2M\;Mean\;\;Ct)$$

**Fig 5:**
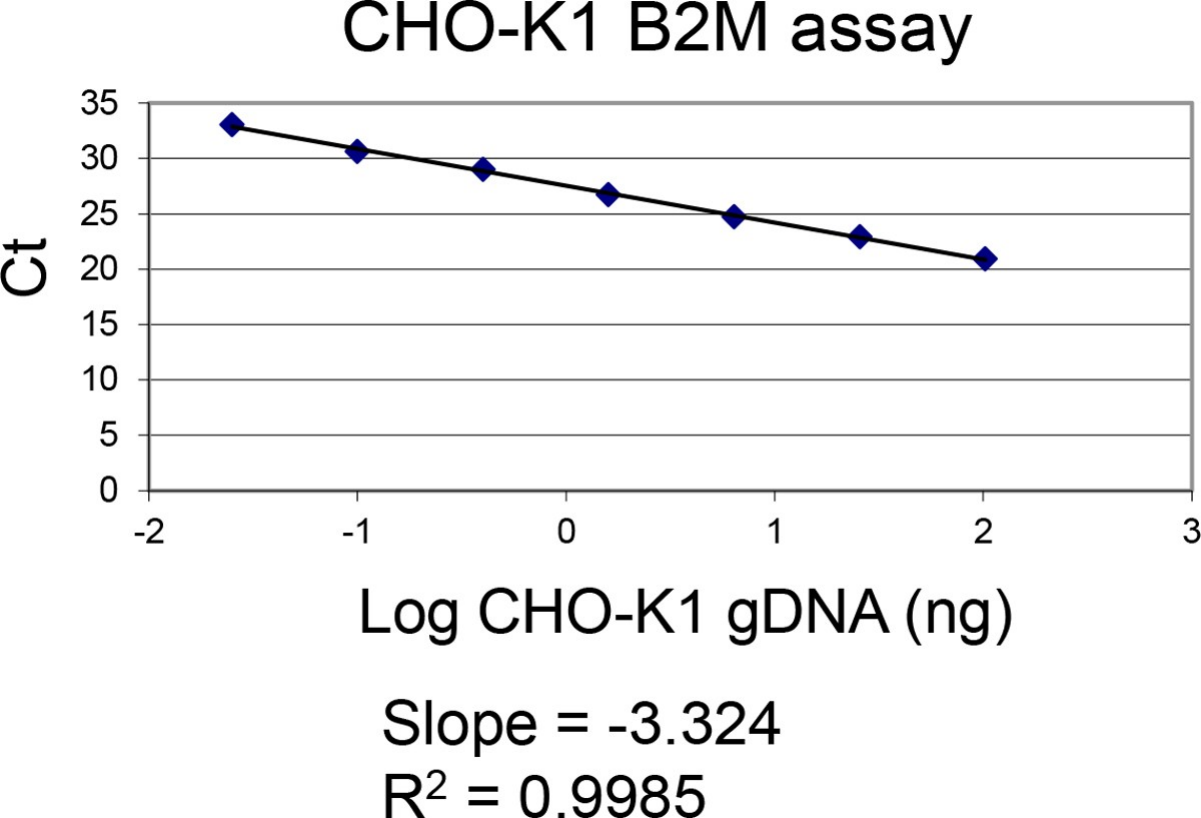
CHO-K1 B2M assay performance on CHO-K1 genomic DNA. The CHO-K1 B2M qPCR assay was tested against a fourfold serial dilution of CHO-K1 genomic DNA spanning 0.025–102.4 ng gDNA. Graphing the Ct versus the log of plasmid copies demonstrates a linear range of 0.025–102.4 ng gDNA with R^2^ values approaching 1 and slope values close to -3.3.

A gene specific primer set was used with a serial dilution of CHO-K1 genomic DNA spanning 0.025–102.4 ng gDNA. This range reflects the quantities of gDNA that are expected to be loaded into the assay. The CHO-K1 B2M assay has acceptable slope and R^2^ values with linearity from 0.025–102.4 ng gDNA (Fig 5).

As all the pylRS/tRNApyl expressing cells are from the same parental cell line, the expectation is that there will be no variation on CHO-K1 B2M copy number and all DNA copy number analyses are reported relative to CHO-K1 B2M. Relative copy number was deemed sufficient to examine how the copy number varied among cell lines. Thus, our stable cell lines were analyzed for pylRS and tRNA genomic copies. The data show that the tRNApyl copy number varies substantially from 62–560 copies while the pylRS had a much narrower range from 4–22 copies (Table 5). As the plasmid used for generating this cell line has a 1:18 ratio of pylRS:tRNApyl we expected this ratio to be observed in our stable cell lines (Table 6). Indeed, this ratio is generally retained in all cell lines suggesting that the integrating plasmids are generally intact. Two exceptions, Clone D and Clone F both show greater tRNApyl copy numbers than expected based on the numbers of pylRS indicating that some cell lines may, in the course of integrating the DNA into the genome, introduce only a portion of the tRNApyl containing plasmid. The tight range of Ct values for CHO-K1 B2M validate the initial assumption that there is no difference in CHO-K1 B2M copies between the cell lines.

**Table 5:** Quantifying gene copy number in genomic DNA.

		tRNApyl			pylRSwt		CHO-K1 B2M	
	Mean	Std Dev	Copy	Mean	Std Dev	Copy	Mean	Std Dev
Cell Line	Ct	Ct	Number	Ct	Ct	Number	Ct	Ct
clone A	19.75	0.16	212.1	23.91	0.03	11.9	27.48	0.08
clone B	21.07	0.08	62.7	24.96	0.02	4.2	27.04	0.05
clone C	20.11	0.18	142.6	24.21	0.11	8.3	27.27	0.06
clone D	18.63	0.22	393.1	23.63	0.10	12.3	27.25	0.04
clone E	19.46	0.10	249.1	23.73	0.07	12.9	27.42	0.10
clone F	18.42	0.24	560.0	23.06	0.01	22.4	27.54	0.16

DNA copy number was determined relative to CHO-K1 B2M.

**Table 6:** Comparing the observed tRNApyl copy number to the predicted tRNApyl copy number.

	pylRSwt	tRNApyl	tRNApyl
Cell Line	observed	observed	predicted
clone A	11.9	212.1	214.2
clone B	4.2	62.7	75.8
clone C	8.3	142.6	149.3
clone D	12.3	393.1	221.7
clone E	12.9	249.1	232.2
clone F	22.4	560.0	402.7

Copy numbers are reported relative to CHO-K1 B2M.

**Fig 6:**
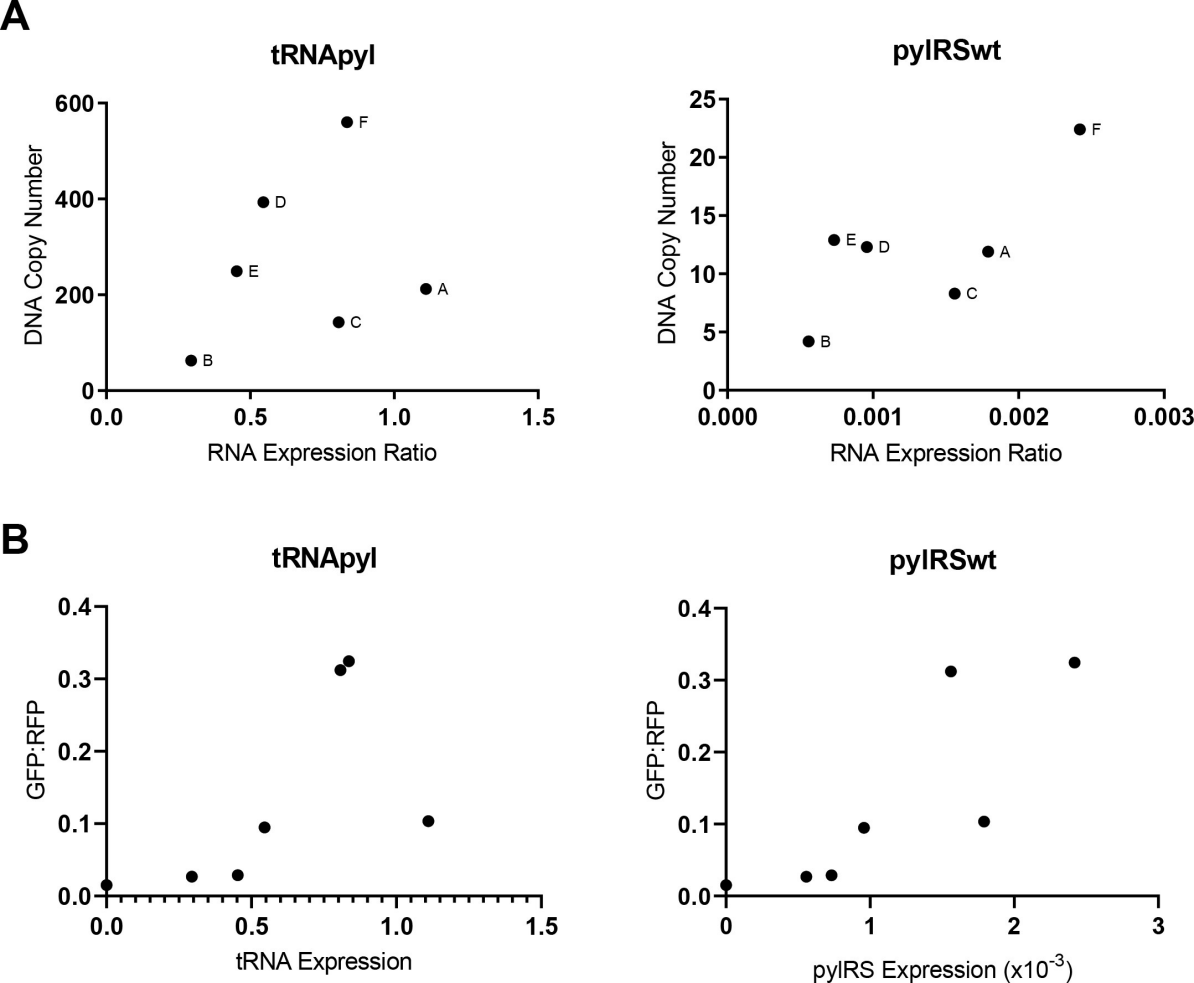
Comparing cell line DNA copy number to RNA expression for tRNApyl and pylRSwt assays. Copy numbers of pylRS and tRNA were determined in six amber suppression competent cell lines. (A) The calculated DNA copy numbers plotted relative to the corresponding RNA expression levels. A loose correlation between copy number and expression is observed for pylRSwt; however, tRNA expression levels do not seem to depend on copy number. (B) The GFP:RFP ratios plotted relative to corresponding RNA expression levels.

We also examined the correlation between DNA copy number and RNA expression level of both tRNApyl and pylRS (Fig 6A). For each cell line the genomic copy number of pylRS and tRNApyl were plotted against their corresponding expression level determine by RT-qPCR. For the most part we observed that cells with increased genetic copy numbers also showed increased expression levels for both pylRS and tRNApyl with notable exceptions. Clones A, B and C showed elevated tRNApyl expression ratios relative to their tRNApyl gene copy numbers; a trend that was also observed with pylRS suggesting that locus of integration may affect expression of these genes.

The biological impact of tRNApyl and pylRS expression on amber suppression efficiency was examined by comparing these levels with the GFP:RFP ratios obtained for each cell line in a functional assay (Fig 3B). the GFP:RFP ratios were determined using the geometric mean data from GFP and RFP by flow cytometry. The GFP levels measure amber suppression while RFP levels represent total expression of the reporter. The ratio of GFP:RFP may be indicative of amber suppression efficacy while accounting for differences in expression of the reporter in the different cell lines. In general, the data shows that higher levels of tRNApyl and pylRS results in increased amber suppression efficacy (Fig 6B and Table 7). It should be noted that transient expression experiments provide a useful, but limited measure of the expression potential of cells due to the short and transient nature of the assay. A better understanding of the true potential of these stable cell lines will need to be assessed using stable expression of very high expressing targets which exceed the capacity of the amber suppression system.

**Table 7:** Comparing cell line RNA expression of tRNApyl and pylRS to GFP:RFP ratio.

	Expression Ratio		Geo Mean		GFP:RFP
Clone	tRNApyl	pylRS	GFP	RFP	ratio
A	1.11E+00	1.79e-03	114	1103	0.103
B	2.95E-01	5.58e-04	82	3054	0.027
C	8.07E-01	1.56e-03	158	506	0.312
D	5.45E-01	9.58e-04	149	1570	0.095
E	4.53E-01	7.32e-04	111	3829	0.029
F	8.36E-01	2.42e-03	148	456	0.325
Neg	ND	ND	14	931	0.015

Expression levels of tRNApyl and pylRS are reported relative to 18S rRNA. Geometric means of GFP and RFP were quantified by flow cytometry. ND, not detected.

Next, we tested the specificity and reproducibility of the assays. To test specificity, we analyzed RNA and gDNA from a parental cell line (not-transfected with tRNApyl or pylRS) and two clones, clones 1 and 2, created by transfecting the parental cell line with tRNApyl and pylRS (S1 Table). The RNA data for tRNApyl and pylRS show expression in the transfected clones but not in the parental line, while the 18S signal is similar across all three cell lines. The gDNA data yields similar results with tRNApyl and pylRS having good signal in the transfected clones and little to no signal in the parental, while the CHO-K1 B2M signal is similar across all three cell lines. The consistent expression of the normalizer genes combined with the differential expression of tRNApyl and pylRS genes demonstrates that the assays are specific for tRNApyl and pylRS.

To demonstrate the reproducibility of this assay, a single prep of RNA or gDNA, isolated from a transfected cell line, was examined in triplicate on three consecutive days using an identical set up (S2 Table). The Mean Ct and Std Dev Ct values for the triplicates on the plate demonstrate the reproducibility within triplicates. The averages and standard deviations were then calculated across all the days and for each assay. The small standard deviation values for all the assays with RNA and gDNA templates demonstrates the reproducibility of these assays.

## Discussion

A vast number of nnAAs have been developed that provide building blocks for protein engineering with unique functional chemistries. This repertoire of nnAAs provides a means for the generation of protein therapeutics with enhanced functionality, and thus hold great promise in producing and potentiating new drugs for the treatment of human disease. However, despite significant advances, the productivity of systems capable of site-specific nnAA incorporation has prevented its widespread use. Genetically encoding nnAA, in both in vitro and in vivo systems, requires the concerted function of an orthogonal aminoacyl tRNA synthetase and its cognate tRNA. In vitro expression systems have successfully addressed many of the limitations, but this methodology requires dedicated cell free expression facilities [8]. On the other hand, cell-based systems have been developed that show potential for producing at levels consistent with manufacturing standards. These can be used with conventional fermentation facilities and methodologies, but require engineered cells, carefully selected for high amber suppression potential.

While it is clear that an orthogonal tRNA synthetase and its cognate tRNA must be expressed by the host cells, the expression levels of these genes required for efficient nnAA incorporation remain largely unknown. This is, in part, due to the difficulties in quantifying tRNA levels which previously relied largely on Northern analyses and more recently on molecular methods which require multiple enzymatic steps [20–22]. Hence, we have developed a qPCR-based method for the quantitation of tRNApyl and pylRS that enable the rapid characterization of cell lines. The assays described here show a > 5 log dynamic range and are effective at quantifying both RNA and gDNA. Engineered cell lines functionally characterized for efficient nnAA incorporation, contained 4–22 copies of pylRS and 65–560 copies of tRNA. More impressively we noted that expression levels of the tRNA approached that of the ribosomal 18S rRNA. This expression level is over 1000-fold greater than pylRS and the endogenous Phe and Met tRNAs. This high level of tRNA may be necessary to compete with release factor (eRF1) for amber codon recognition. eRF1 mediates the process of protein termination by recognizing stop codons and promoting the release of the polypeptide chain by the ribosome [23–24]. Thus, high levels of tRNApyl are likely needed to compete for amber codon binding and readthrough. Indeed, cells with higher levels of pylRS and tRNA expression show improved amber suppression efficacy using a transiently expressed reporter construct. However, it should be noted that the assay provides a relative quantitation of tRNApyl and pylRS expression levels which is useful for the discrimination of cell lines. That said, analyses of technical replicates show consistent results suggesting that the qPCR method is both robust and reproducible (S2 Table).

Beyond quantifying the tRNA, qPCR allows us to examine the extent of tRNApyl processing. In our cells tRNApyl expression is under control of a PolIII promoter that regulates the expression of a transcript that requires both 3’ and 5’ processing that is required to generate functional tRNApyl. The assays developed here discriminate between unprocessed and mature (functional) tRNApyl allowing for an assessment of tRNA functionality. In the cells tested we observed ~90% mature tRNA indicating that processing of this tRNA is efficient despite the high expression levels. Overall, the methodology described provides us with a tool for the characterization of cells containing aaRS/tRNA based systems as well as a key metric for cell line selection. The analysis of six cell lines showed a relationship between copy number and expression levels. Two notable exceptions for tRNApyl indicate that site of integration influences expression of these genes and highlights that copy number alone is not a predictive metric for tRNA expression. In addition, we show that increased amber suppression efficacy is observed in cells with higher levels of the pylRS/tRNApyl. Future studies with additional cell lines or other platforms are needed to better establish this relationship. As we develop new engineered cell lines the minimal requirements of tRNA and pylRS for efficient nnAA incorporation at >1g/L yields of target will become evident. One area of import is the genetic stability of the pylRS/tRNApyl system which must support efficient amber suppression over 70 generations in a manufacturing setting. The assay developed here is an invaluable tool in assessing the genetic stability of the system considering the highly repetitive nature of the tRNApyl cassettes and their potential for recombination. Overall the assay is readily applicable to the quantitation of exogenous aaRS/tRNAs and offers a tool to better understand the limitations of these nnAA incorporation systems.

## Supporting information

S1 Table Data table examining the specificity of the qPCR assays. (DOCX)

S2 Table Data table examining the reproducibility of the qPCR assays. (DOCX)
