# Supplementary material for: qPCR assays to quantitate tRNApyl and pylRS expression in engineered cell lines
Source: PLoS One. 2019 May 9;14(5):e0216356. doi: 10.1371/journal.pone.0216356 (PMC6508675; doi:10.1371/journal.pone.0216356)
Supplement: S1 Table — (DOCX) [file pone.0216356.s001.docx]

**S1 Table. qPCR assay specificity**

| **A** |  |  |  |  |  |  |  |  |
| --- | --- | --- | --- | --- | --- | --- | --- | --- |
|  |  |  | **tRNApyl** |  |  | **tRNApyl unprocessed** | | |
|  |  | Mean | Std Dev | Expression |  | Mean | Std Dev | Expression |
| **Cell Line** |  | Ct | Ct | Ratio |  | Ct | Ct | Ratio |
| Parental |  | ND | ND | ND |  | ND | ND | ND |
| Clone 1 |  | 21.11 | 0.14 | 9.31E-03 |  | 23.41 | 0.06 | 1.90E-03 |
| Clone 2 |  | 20.27 | 0.15 | 1.47E-02 |  | 21.79 | 0.10 | 5.16E-03 |
|  |  |  |  |  |  |  |  |  |
|  |  |  |  |  |  |  |  |  |
|  |  |  | **pylRSwt** |  |  | **18s** | |  |
|  |  | Mean | Std Dev | Expression |  | Mean | Std Dev |  |
| **Cell Line** |  | Ct | Ct | Ratio |  | Ct | Ct |  |
| Parental |  | ND | ND | ND |  | 14.40 | 0.11 |  |
| Clone 1 |  | 27.53 | 0.11 | 1.09E-04 |  | 14.37 | 0.12 |  |
| Clone 2 |  | 25.40 | 0.07 | 4.22E-04 |  | 14.19 | 0.07 |  |
|  |  |  |  |  |  |  |  |  |

| **B** |  |  |  |  |  |  |  |  |  |  |  |
| --- | --- | --- | --- | --- | --- | --- | --- | --- | --- | --- | --- |
|  |  |  | **tRNApyl** |  |  |  | **pylRSwt** |  |  | **CHO-K1 B2M** | |
|  |  | Mean | Std Dev | Copy |  | Mean | Std Dev | Copy |  | Mean | Std Dev |
| **Cell Line** |  | Ct | Ct | Number |  | Ct | Ct | Number |  | Ct | Ct |
| Parental |  | 38.35 | 0.31 | 0.0002 |  | ND | ND | ND |  | 26.34 | 0.08 |
| Clone 1 |  | 22.73 | 0.15 | 13.17 |  | 28.21 | 0.10 | 0.29 |  | 26.44 | 0.06 |
| Clone 2 |  | 22.29 | 0.12 | 19.48 |  | 27.45 | 0.15 | 0.54 |  | 26.57 | 0.12 |

Samples from non-transfected parental cells and transfected clones 1 and 2 were analyzed for RNA expression (A) and copy number (B). The normalizer genes, 18S (A) and CHO-K1 B2M (B), had similar signal across all three cell lines. The tRNApyl and pylRS only had signal in the transfected clones demonstrating that the assays are specific. ND, not detected.
