# Supplementary material for: qPCR assays to quantitate tRNApyl and pylRS expression in engineered cell lines
Source: PLoS One. 2019 May 9;14(5):e0216356. doi: 10.1371/journal.pone.0216356 (PMC6508675; doi:10.1371/journal.pone.0216356)
Supplement: S2 Table — (DOCX) [file pone.0216356.s002.docx]

**S2 Table. qPCR assay reproducibility**.

| **A** |  |  |  |  |  |  |  |  |  |
| --- | --- | --- | --- | --- | --- | --- | --- | --- | --- |
|  |  |  | **tRNApyl** |  |  | **tRNApyl unprocessed** | | |  |
|  |  | Mean | Std Dev | Expression | | Mean | Std Dev | Expression | |
|  |  | Ct | Ct | Ratio |  | Ct | Ct | Ratio |  |
| Day 1 |  | 15.01 | 0.08 | 34.67 |  | 19.09 | 0.06 | 2.06 |  |
| Day 2 |  | 15.37 | 0.15 | 33.49 |  | 19.22 | 0.08 | 2.32 |  |
| Day 3 |  | 15.43 | 0.13 | 32.02 |  | 19.32 | 0.11 | 2.17 |  |
| Average |  | 15.27 |  | 33.40 |  | 19.21 |  | 2.18 |  |
| Std Dev |  | 0.23 |  | 1.33 |  | 0.12 |  | 0.13 |  |
|  |  |  |  |  |  |  |  |  |  |
|  |  |  |  |  |  |  |  |  |  |
|  |  |  | **pylRSwt** |  |  | **18s** | |  |  |
|  |  | Mean | Std Dev | Expression | | Mean | Std Dev |  |  |
|  |  | Ct | Ct | Ratio |  | Ct | Ct |  |  |
| Day 1 |  | 24.73 | 0.07 | 4.11E-02 |  | 20.13 | 0.23 |  |  |
| Day 2 |  | 24.74 | 0.07 | 5.07E-02 |  | 20.44 | 0.00 |  |  |
| Day 3 |  | 24.86 | 0.12 | 4.65E-02 |  | 20.43 | 0.17 |  |  |
| Average |  | 24.78 |  | 4.61E-02 |  | 20.33 |  |  |  |
| Std Dev |  | 0.07 |  | 4.83E-03 |  | 0.18 |  |  |  |

| **B** |  |  |  |  |  |  |  |  |  |  |  |
| --- | --- | --- | --- | --- | --- | --- | --- | --- | --- | --- | --- |
|  |  |  | **tRNApyl** |  |  |  | **pylRSwt** |  |  | **CHO-K1 B2M** | |
|  |  | Mean | Std Dev | Copy |  | Mean | Std Dev | Copy |  | Mean | Std Dev |
|  |  | Ct | Ct | Number |  | Ct | Ct | Number |  | Ct | Ct |
| Day 1 |  | 18.36 | 0.16 | 339.2 |  | 23.45 | 0.18 | 10.0 |  | 26.77 | 0.09 |
| Day 2 |  | 19.55 | 0.06 | 292.4 |  | 24.30 | 0.05 | 10.9 |  | 27.75 | 0.03 |
| Day 3 |  | 18.97 | 0.11 | 371.6 |  | 24.01 | 0.06 | 11.2 |  | 27.50 | 0.10 |
| Average |  | 18.96 |  | 334.40 |  | 23.92 |  | 10.72 |  | 27.34 |  |
| Std Dev |  | 0.60 |  | 39.78 |  | 0.43 |  | 0.63 |  | 0.51 |  |

A nucleic acid preparation from cells transfected with the pylRS/tRNApyl expression constructs was assayed in triplicate on three separate days for RNA expression (A) and copy number (B). The low standard deviation values both within plates and across days shows the reproducibility of the assays. tRNA and pylRS expression ratios are reported relative to 18S rRNA. Copy number analysis are shown relative to CHO-K1 B2M.
